# Adaptive Skeletal Muscle Action Requires Anticipation and “Conscious Broadcasting”

**DOI:** 10.3389/fpsyg.2012.00369

**Published:** 2012-09-28

**Authors:** T. Andrew Poehlman, Tiffany K. Jantz, Ezequiel Morsella

**Affiliations:** ^1^Marketing Department, Cox School of Business, Southern Methodist UniversityDallas, TX, USA; ^2^Department of Psychology, San Francisco State UniversitySan Francisco, CA, USA; ^3^Department of Neurology, University of CaliforniaSan Francisco, CA, USA

**Keywords:** consciousness, skeletal muscle, anticipation, ideomotor action, voluntary action

## Abstract

Historically, the conscious and anticipatory processes involved in voluntary action have been associated with the loftiest heights of nervous function. Concepts like mental time travel, “theory of mind,” and the formation of “the self” have been at the center of many attempts to determine the purpose of consciousness. Eventually, more reductionistic accounts of consciousness emerged, proposing rather that conscious states play a much more basic role in nervous function. Though the widely held *integration consensus* proposes that conscious states integrate information-processing structures and events that would otherwise be independent, *Supramodular Interaction Theory* (SIT) argues that conscious states are necessary for the integration of only certain kinds of information. As revealed in this selective review, this integration is related to what is casually referred to as “voluntary” action, which is intimately related to the skeletal muscle output system. Through a peculiar form of broadcasting, conscious integration often controls and guides action via “ideomotor” mechanisms, where anticipatory processes play a central role. Our selective review covers evidence (including findings from anesthesia research) for the integration consensus, SIT, and ideomotor theory.

Understanding how consciousness arises from the brain is a far greater task than what the average person might surmise. The unfortunate truth is that, at the present stage of understanding, not only do scientists not have a clue regarding how conscious states emerge from the human nervous system, but they do not even possess the smallest inkling regarding how something like consciousness could emerge from any set of real or hypothetical circumstances (Levine, 1983; Banks, 1995; Godwin et al., in press). As Shallice (1972, p. 383) concludes, “The problem of consciousness occupies an analogous position for cognitive psychology as the problem of language behavior does for behaviorism, namely, an unsolved anomaly within the domain of the approach.”

In this selective review, we discuss a subset of findings revealing some humble progress regarding this puzzle. This progress stems primarily from observations of everyday action planning, anticipatory processing, and the voluntary control of overt action through the skeletal muscle system. Examination of these interconnections reveals why, for every voluntary action, the actor can self-report conscious content responsible for that action. As explained below, the implications of this often overlooked but reliable observation (that voluntary actions are connected to conscious content) are important, even if self-reports on the causes of these actions by actors are often inaccurate (Nisbett and Wilson, 1977; Wegner, 2002, 2003). By integrating various disparate literatures, we put a non-traditional frame on the connections among anticipation, conscious states, and skeletal muscle action (“skeletomotor action,” for short). For instance, instead of studying consciousness by focusing on perception (the dominant approach; Crick and Koch, 2003), we examine consciousness by working backward from overt action to trace the central processes responsible for action (Morsella and Bargh, 2010). We also find relevant clues about the nature of consciousness from research on anesthesia.

Prior to discussing the interconnections among conscious states, anticipation, and skeletomotor action, it is important to explain what we mean by the generally ethereal concept of consciousness. “Consciousness,” which is also sometimes referred to as “sentience” (Pinker, 1997), a “phenomenal state” (Jackson, 1982; Tye, 1999), “qualia” (Gray, 2004), or “subjective experience,” has been perhaps best defined by the philosopher Nagel (1974), who proposed that an organism possesses subjective experiences if there is *something it is like* to be that organism – something it is like, for example, to be human and experience warmth, love, yellowness, or breathlessness. Similarly, Block (1995, p. 227) says, “the phenomenally conscious aspect of a state is what it is like to be in that state.” In this article, we are interested in this most basic form of consciousness, a form of consciousness that should be distinguished from higher forms of consciousness (e.g., self-consciousness, consciousness of one’s culture, etc.). From our perspective, if any thing has an experience of any kind, then it possesses the kind of consciousness in which we are interested.

While it is true that throughout the history of psychology, “consciousness” has been coupled with outstandingly complex phenomena like “the self” and mental time travel (see review of high-level theories in Morsella, 2005), recently, less lofty accounts of consciousness have emerged, proposing rather that conscious states play a much more basic role in nervous function. One promising direction of this research has been achieved by juxtaposing conscious and unconscious processes in terms of their cognitive and neural correlates (e.g., Shallice, 1972; Baars, 1988, 2002; Logothetis and Schall, 1989; Crick and Koch, 1995; Kinsbourne, 1996; Wegner and Bargh, 1998; Grossberg, 1999; Di Lollo et al., 2000; Dehaene and Naccache, 2001; Gray, 2004; Libet, 2004; Laureys, 2005; Morsella, 2005; Merker, 2007; Doesburg et al., 2009; Damasio, 2010; Boly et al., 2011). This contrastive approach has revealed that many sophisticated processes can, and do, occur unconsciously (cf., Godwin et al., in press). For example, motor programming – which calculates the muscle fibers that should be activated at a given time in order to enact action – falls into the category of processes that can occur unconsciously (James, 1890; Grossberg, 1999; Fecteau et al., 2001; Rossetti, 2001; Rosenbaum, 2002; Goodale and Milner, 2004; Johnson and Haggard, 2005; Heath et al., 2008). Additionally, low-level (or “pre-conscious”) perceptual processing also occurs unconsciously (Crick and Koch, 1995; Gray, 2004; Koch, 2004). Other mechanisms linking perception to action can also transpire unconsciously, as with the relatively obvious case of reflexes or in the less common case of automatisms (see review in Morsella and Bargh, 2011). It is important to note, that subliminal stimuli have been shown to reliably elicit motor acts as well (Fehrer and Biederman, 1962; Fehrer and Raab, 1962; Taylor and McCloskey, 1990, 1996; Hallett, 2007).

This contrastive approach has revealed that so much of nervous function is both unconscious and sophisticated. It has lead many researchers to what would have once been an unanticipated question: What do conscious states, in fact, add to brain function?

At present, it seems the answer lies in what has come to be called the *integration consensus* (Tononi and Edelman, 1988; Damasio, 1989; Freeman, 1991; Baars, 1998; Zeki and Bartels, 1999; Dehaene and Naccache, 2001; Llinás and Ribary, 2001; Varela et al., 2001; Clark, 2002; Ortinski and Meador, 2004; Sergent and Dehaene, 2004; Del Cul et al., 2007; Doesburg et al., 2009; Ulhaas et al., 2009; Boly et al., 2011). The integration consensus proposes that conscious states integrate neural activities and information-processing structures that would otherwise be independent (see a review in Baars, 2005). For example, when actions are decoupled from consciousness (e.g., in neurological disorders such as anarchic hand syndrome and utilization behavior syndrome; Lhermitte, 1983; Marchetti and Della Sala, 1998), the actions (e.g., a hand meandering through irrelevant actions like tugging at its owner’s shirt) often appear impulsive or inappropriate, as if they are not influenced by the kinds of information by which they should be influenced (Morsella and Bargh, 2011). Most theoretical frameworks in the integration consensus speak of conscious information as being available “globally” in some kind of mental workspace (Baars, 2002; Sergent and Dehaene, 2004).

Separate from this global-reach system of conscious integration, unconscious processes involve smaller networks of brain areas and require less widespread activation than their conscious counterparts (Sergent and Dehaene, 2004; Baars, 2005; Gaillard et al., 2009). (See review in Morsella et al., 2010.) For example, the unconsciously mediated action of reflexive swallowing involves substantially fewer brain regions than volitional swallowing (Kern et al., 2001; Ortinski and Meador, 2004). Additionally, in the unconscious phases of deep sleep, auditory input yields activity that is limited to only the primary auditory cortex (Portas et al., 2000).

It seems that, for consciousness, the mode of interaction among regions is as important as the nature and loci of the regions (Buzsáki, 2006). For instance, the presence or lack of what has been called “interregional synchrony” leads to different cognitive and behavioral outcomes (Hummel and Gerloff, 2005; see review of neuronal communication through “coherence” in Fries, 2005). In binocular rivalry, for example, it is evident that the mode of interaction between areas is important for conscious states. During this phenomenon (Logothetis and Schall, 1989), an observer is presented with different visual stimuli to each eye simultaneously (e.g., an image of a house in one eye and of a face in the other). It might seem reasonable that, faced with such stimuli, an observer would perceive an image combining both objects – a house overlapping a face. Surprisingly, even though both images are always present, an observer experiences seeing only one object at time (i.e., a house and then a face). At any moment, the observer is unaware of the computational processes leading to this outcome; the conflict and mechanism of resolution are unconscious. Neurally, while experiencing binocular rivalry, it is only the conscious percept that is coupled, in terms of interregional synchrony, to both perceptual brain activity and motor-related processes in frontal cortex, thus supporting the view that the mode of interaction between areas, and not just activation of the areas, is important for consciousness (Doesburg et al., 2009).

## Evidence from research on anesthesia

Supporting the integration consensus, findings in the field of anesthesiology suggest that anesthetic agents work on consciousness in part by halting the integration of information across widespread brain networks (Mashour, 2004; Hudetz, 2006; Alkire et al., 2008; Lee et al., 2009). Anesthetics may inhibit integration by acting on structures that are necessary for widespread cortical broadcasting and by slowing neural responses, thereby affecting synchronization (Munglani et al., 1993; Alkire et al., 2008). Indeed, Flohr’s (1995) *information-processing theory*, John and Prichep’s (2005) *anesthetic cascade*, Mashour’s (2004) *cognitive unbinding paradigm*, and Alkire et al.’s (2000) *unified theory of narcosis* all directly or indirectly support the idea that anesthetics are acting by disrupting integration in the brain (Mashour, 2006).

Regarding thalamic accounts of consciousness (e.g., Penfield and Jasper, 1954; Merker, 2007), the most consistently reported effect of anesthetic agents is the reduction in thalamic blood flow and metabolism during the loss of consciousness (Hudetz, 2006; Alkire et al., 2008; Långsjö et al., 2012). It has also been suggested that thalamic blocking of somatosensory information may be the cause of the anesthetic state (Angel, 1991; Hudetz, 2006). Some anesthetics may work by affecting the posterior lateral corticothalamic complex and perhaps a medial cortical core, either directly or indirectly, thus resulting in unconsciousness (Alkire et al., 2008). Additionally, thalamocortical connectivity is associated with recovery from vegetative states (Laureys et al., 2000a,b; Mashour, 2006). However, not all anesthetics act on the thalamus in the same manner. The anesthetic ketamine, for example, results in increases in thalamic metabolism while sevoflurane sedation decreases such metabolism while the subject remains conscious (cf., Alkire et al., 2008). Additionally, studies using electroencephalography (EEG) have shown that, as soon as a subject loses consciousness, there is a marked change in cortical EEG, while thalamic EEG remains relatively the same for some minutes afterward. This begs the question as to whether the thalamus is inactivated directly or perhaps indirectly following cortical suppression (Alkire et al., 2008).

Some research on anesthesia suggests that frontal cortex alone may not constitute consciousness (Penfield and Jasper, 1954; Merker, 2007; Alkire et al., 2008). For instance, recent investigations into feedforward and feedbackward connectivity while under anesthesia suggest that conscious states are associated with, not only frontal activations, but specific frontoparietal networks (Ku et al., 2011). Additionally, low doses of anesthetics have been shown to slow the feedback stream of cortical processing, while increasing doses slow both the feedforward and feedback streams of cortical processing. These findings suggest that some form of widespread feedback dynamics, or “reentrant” processing (Di Lollo et al., 2000; Fahrenfort et al., 2007), may play an integral part in conscious awareness (see below; Hudetz, 2006; Långsjö et al., 2012). In addition, the notion that frontal cortex is unnecessary for consciousness is consistent with investigations on prefrontal lobe syndromes (Gray, 2004), the phenomenology of action and behavior (Desmurget et al., 2009; Desmurget and Sirigu, 2010), and the psychophysiology of consciousness in dreams, which involves prefrontal deactivations (Muzur et al., 2002). (See evidence for a necessary role of frontal cortex in consciousness in Boly et al., 2011). There are other regions that may be unnecessary for the brain to constitute a basic form of consciousness. For example, although the absence of the spinal cord or cerebellum leads to sensory, motor, cognitive, and affective deficits, the non-participation of these regions does not seem to eliminate basic consciousness (Schmahmann, 1998; Morsella et al., 2010). Similarly, non-participation of the basal ganglia, hippocampus, mammillary bodies, right cerebral cortex, or mediodorsal nucleus of the thalamus does not seem to hinder the ability of the nervous system to generate a basic form of consciousness (see evidence in Morsella et al., 2010; Godwin et al., in press).

## Supramodular interaction theory and limitations of the integration consensus

One limitation of the integration consensus is that it fails to specify exactly which kinds of integration require conscious states and which kinds can occur unconsciously. For example, conscious processing is unnecessary for integrations across different sensory modalities (e.g., the binding of features in perceptual objects) or integrations involving smooth muscle effectors (e.g., integrations in the pupillary reflex; Morsella et al., 2009a). In both cases, these integrations/conflicts can transpire unconsciously. In contrast, people tend to be aware of some of the conflicts in their nervous system. When a swimmer holds her breath underwater, for example, she cannot help but be aware of the conflict of restraining an automatic process like breathing. Further, *approach–approach* conflicts also beg for awareness (Lewin, 1935; Miller, 1959). These types of conflicts, *conscious conflicts* (Morsella, 2005), involve competition for control of the skeletal muscle output system and are triggered by incompatible skeletomotor plans, as when one holds one’s breath while underwater, suppresses uttering something, or inhibits a prepotent response in a laboratory *response interference paradigm* (e.g., the Stroop and Flanker tasks; Stroop, 1935; Eriksen and Eriksen, 1974). *Supramodular Interaction Theory* (SIT; Morsella, 2005) proposes that, while the primary function of conscious states is to integrate information, only certain kinds of information require conscious integration. Specifically, it is high-level information in the service of curbing skeletomotor action so that such action is adaptive, as in the case of holding one’s breath or breathing at a faster rate for some reward. Conscious conflicts are a dramatic case of such interactions. (The theory is called “supramodular,” because the integrations occur at a high-level, beyond that of the Fodorian module, which is used for, say, color, and motion detection; the term “interaction” is used in the theory because conscious states permit interactions between high-level systems vying for skeletomotor control; see treatments of modularity in Fodor, 1983; Callebaut and Rasskin-Gutman, 2009.) The actual integration amongst such *response systems* may actually be “post-conscious” (Morsella, 2005). (For a thorough review of the nature of the difference between the *access* of information during conscious states and the *subjectivity* associated with that information, see Atkinson et al., 2000.) From our standpoint, conscious states are necessary, not to integrate perceptual-level processes (like feature binding), but to permit interactions among action goal inclinations that, eventually, influence the skeletal muscle system; this idea is captured in the principle of *Parallel Responses into Skeletal Muscle* (PRISM; Morsella, 2005).

To summarize in different and more concrete terms, SIT proposes that, in the nervous system, there are three distinct kinds of integration or “binding” (Morsella and Bargh, 2011). Perceptual binding (or *afference binding*) is the binding of perceptual processes and representations. This occurs in feature binding (e.g., the binding of shape to color; Zeki and Bartels, 1999) and intersensory binding (McGurk and MacDonald, 1976; Vroomen and de Gelder, 2003), in which disparate senses integrate information across the perceptual field (e.g., visual and auditory inputs regarding the source of a sound interact unconsciously). (See additional evidence for unconscious afference binding in Zmigrod and Hommel, 2011).

The second form of binding (*efference binding*) links perceptual processing to action/motor production (Haggard et al., 2002). (For advanced treatments of the topic of integration across perception and action, see Hommel et al., 2001; Astor-Jack and Haggard, 2005; Magen and Cohen, 2010.) This kind of stimulus-response (*S* → *R*) binding allows for automatic button presses in response to a cue. Research has shown that efference binding can happen unconsciously, as when subjects are able to select the correct motor response (one of two button presses) when confronted with a subliminal cue (Fehrer and Biederman, 1962; Fehrer and Raab, 1962; Taylor and McCloskey, 1990, 1996; Hallett, 2007). (For studies revealing how instructions held in mind can lead to *S* → *R* mappings that resemble that of reflexes, see Cohen-Kdoshay and Meiran, 2009; Hommel, 2000; Wenke et al., 2007). More commonly, this kind of binding can also be mediated unconsciously in actions such as the pain withdrawal reflex and reflexive swallowing and inhalation. The third form of binding, *efference–efference binding*, occurs when two streams of efference binding are trying to influence skeletomotor action simultaneously (Morsella and Bargh, 2011). Importantly, these streams of efference are “bound” at, at least, the level of overt action. For instance, when a swimmer holds her breath, she experiences the conflict between the two efferent streams (wanting to inhale/wanting to suppress inhalation) and produces an action that is a “binding” of the two inclinations. In this case, the integration at the level of overt behavior is her holding her breath but behaving less comfortably than if provided with oxygen. In such a way, conflicted behavior is overtly different from non-conflicted behavior, as Skinner notes (Skinner, 1953). To him, such behaviors are more perturbable and slower in their execution. In the context of laboratory research, conflicted skeletomotor action is also apparent when a research participant suppresses a prepotent response like word reading in a response interference paradigm such as the classic Stroop task (where participants are asked only to name the color in which a word is presented). Importantly, conflicts involving perceptual processing or smooth muscle do not yield such changes in consciousness (Morsella et al., 2009a).

The tenets of SIT principally concern, not which kinds of interactions do and do not occur with phenomenal mediation, but, in identifying the function of consciousness, which kinds of basic processes cannot occur without phenomenal mediation. Thus, it is not within the scope of SIT to identify all the modular or supramodular outputs that one can be conscious of. Rather, SIT is about which integrative processes require conscious mediation. During conflicts that require conscious mediation, one is aware of the conflicting components (e.g., pain and hunger) that are brought together to influence action. Interestingly, however, one is unaware of the computational products of conscious interaction, which, should they exist, are observable only in the form of expressed behavior (e.g., breathing faster for some reward; Morsella, 2005). In other words, one is unconscious of the representations reflecting the resolution of the conflict (if such representations exist). Consciousness is necessary for the integration, but the integration is best represented, not in consciousness, but in overt behavior. Hence, our theoretical approach is named “SIT” and not “supramodular *integration* theory,” because, for the reasons just outlined, the term *integration* is a loaded term. One must consider that “to combine” does not necessarily imply “to resolve.”

It should be reiterated that this survey comprises a selective review of research findings, a review based on one specific vantage point (for other accounts of information integration, see Tononi and Edelman, 1988; Logan et al., 1999; Baars, 2002; Miller and Ulrich, 2003; Goodale and Milner, 2004; Dijksterhuis and Nordgren, 2006; Ulrich et al., 2007). From the present standpoint, consciousness can be construed as a “crosstalk” medium that allows conflicting efference streams to influence action collectively, leading to *integrated actions* (Morsella and Bargh, 2011) such as our swimmer holding her breath. Absent consciousness, behavior can be influenced by only one of the efference streams, leading to *un-integrated actions* (Morsella and Bargh, 2011) such as unconsciously inhaling while underwater, or, in another common example, reflexively dropping a carelessly made latte at Starbucks, because it feels too hot. As mentioned above, the integration afforded by consciousness involves high-level information that can be polysensory, and occurs at a stage of processing “beyond” that of the traditional Fodorian module (Fodor, 1983). The information that is represented consciously (or, in the “conscious field”; Morsella, 2005) can be considered the output of systems that are usually consciously impenetrable: In this sense, one may be able to suppress dropping the latte, but one cannot suppress the subjective urge to perform the act. From this standpoint, conscious crosstalk permits important information (or outputs) to be broadcasted to the systems responsible for skeletomotor action.

In summary, the difference between unconscious action (i.e., reflexes and the like) and conscious action is that the former is always a case of un-integrated action, and the latter *can* be a case of “integrated action.” Our central claim here is that integrated action occurs when two (or more) action plans – that might normally influence behavior on their own – simultaneously co-activate and try to influence the same skeletal muscle effector at the same moment in time (Morsella and Bargh, 2011). It follows then that integrated action in every day life occurs when one: *holds* one’s breath, *refrains* from dropping a hot latte, *does not* scratch an itch, or breathes *faster than normal* on purpose (e.g., for some reward). In ours and others’ academic studies, integrated actions occur when participants are asked to do things like suppress a prepotent response in a laboratory paradigm such as the Stroop Task. (See Morsella et al., 2011, for a quantitative review of laboratory evidence supporting SIT.)

## The skeletal muscle effector system

The skeletal muscle effector system differs substantively from most effector systems in the body (e.g., smooth muscle) in that distinct brain regions and brain systems try to control it in different – and often opposing – ways. From this standpoint, skeletal muscle is like a single steering wheel controlled simultaneously by multiple agentic systems. Each of these agentic systems has its own particular operating principles, phylogenetic origins, and concerns. While motor programs are instantiated by unconscious algorithms (Rosenbaum, 2002), the selection of higher level action *goals* happens because conscious states are able to crosstalk, which in turn leads to constraint and curbing of skeletomotor output. For example, one system in a chef’s body “protests” when she accidentally touches a hot pot in her kitchen, but another system reinforces another act just as accidental when she mindlessly brings sugar to her lips in a moment of thought. As in the case of our chef, people are conscious of the tendencies (e.g., the urges and cravings) of these systems, but not necessarily of the factors engendering the tendencies themselves (tissue damage versus the relative rarity of sugar in nature; Nisbett and Wilson, 1977; Baker et al., 2004).

It has been known since at least the nineteenth century that skeletal muscle (or “striated muscle”) is the only bodily effector system that *can be* (though often it is not) controlled consciously. However, why this is so has never been addressed theoretically. SIT is – in essence – a systematic reinterpretation of this age-old fact: *Skeletomotor actions are at times “consciously mediated” because these actions are directed by multiple, encapsulated systems that require conscious states to crosstalk and yield adaptive action, especially when the systems are in conflict* (Morsella, 2005). Although identifying still higher level systems is beyond the present purview of SIT, PRISM has correctly predicted that certain aspects of emotional behaviors, reproductive behaviors, parental care, and addiction-related behaviors should be coupled with conscious states, because they all exert influence over skeletal muscle plans.

It should be emphasized that there is nothing intrinsically special about skeletal muscle that causes it to be related to conscious states. Conscious processing distinguishes itself from unconscious processing not simply because it involves skeletal muscle, but because of the particular *way* conscious processing involves skeletal muscle: encapsulated systems in the brain vie to implement their own concerns over the organism in the arena of skeletomotor action planning. Yet, it is important to keep in mind that skeletal muscle is often controlled without conscious mediation, like when a person reading an academic paper shifts his posture, blinks, breathes, or yawns.

## Marrying consciousness to the anticipatory, physiological system it subserves

A primary strength of this approach is that, instead of trying to reverse engineer the purpose of consciousness by examining all that consciousness is capable of doing, it integrates consciousness with most basic of physiological processes it evolved to subserve. From this view, consciousness is one of many processes in the service of adaptive skeletomotor control, which is not surprising given that the primary function of the entire nervous system is to activate the right muscles at the right time. Richard Dawkins notes this succinctly, “The main way in which brains actually contribute to the success of survival machines is by controlling and coordinating the contraction of muscles” (Dawkins, 1976, p. 49). And Roe and Simpson (1958) propose that, in evolutionary history, overt action is *the* critical product of a nervous system, because natural selection can operate only on overt action.

**Figure 1:**
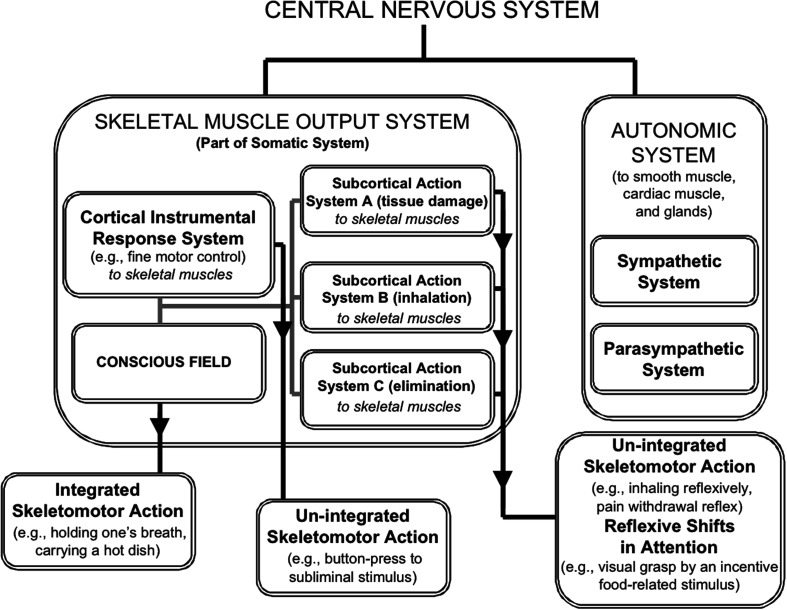
**The major divisions of the nervous system and the circumscribed province of conscious processing within the system**. The major divisions include the Somatic and Autonomic systems. Within the former, Fodorian modules operate within a few multimodal, supramodular response systems (the cortical *instrumental system* and subcortical action systems), each defined by its concern (e.g., tissue damage and elimination). The instrumental system can control fine motor acts through ideomotor processing. Afference binding within systems can be unconscious. Although response systems can influence action directly, as in the case of un-integrated actions, only in virtue of conscious states can multiple response systems interact and influence action collectively, as when one holds one’s breath.

Our approach outlines how consciousness is a phenomenon falling squarely within the *somatic nervous system* (Figure 1); it is within the somatic system that instrumental actions (e.g., holding a hot cup of coffee) are achieved through the mysterious phenomenon of *direct cognitive control* (Morsella et al., 2009c). Interestingly, direct cognitive control is probably best exemplified by one’s ability to immediately control the direction of thought or the movements of a finger or arm (or any other skeletal muscle effectors). Further, when *direct control* is unavailable, indirect forms of control can be implemented. For example, while it is clear that one may not be able to directly influence one’s affective/incentive states at will (Öhman and Mineka, 2001), a nurse can watch her favorite comedy to cheer herself up after a trying day watching people suffer. In other words, regarding direct cognitive control, no one can make oneself intentionally become frightened, happy, angry, sad, or become hungry if the adequate conditions are absent. Yet, people use indirect cognitive control to seek and even pay for certain experiences (e.g., going to movies or comedy clubs) to put themselves in a desired state that cannot be instantiated through an act of will.

While instrumental use of the skeletomotor system involves direct cognitive control, an additional component is often required: a mental representation of the instrumental consequences of action. For instance, we must have the idea of what a cup of coffee looks or feels like in our hands in order to perform this type of instrumental action. Because of this, the skeletomotor system is – by nature – highly anticipatory (Frith et al., 2000; Berthoz, 2002; Llinás, 2002). The operating principles of the directed actions of this system are perhaps best understood in terms of the historical notion of *ideomotor processing* (Greenwald, 1970; Hommel et al., 2001; Hommel, 2009; Hommel and Elsner, 2009). Ideomotor theory holds that the mental image of an instrumental action tends to lead to the execution of that action (Lotze, 1852; Harleß, 1861; James, 1890), with the motor programming involved being unconscious (James, 1890). Simply imagining moving your right arm to reach out for that coffee cup makes the action more likely to occur (for a treatment of why the motor programs involved are unconscious, see Gray, 1995, 2004; Grossberg, 1999; Prinz, 2003). Originating in the times of Lotze (1852), Harleß (1861), and Carpenter (1874), the hypothesis states that action guidance and action knowledge are limited to perceptual-like representations (or, *event codes*; cf., Hommel et al., 2001) of action outcomes (e.g., the “image” of one’s finger flexing; Gray, 1995, 2004; Rossetti, 2001; Rosenbaum, 2002; Jeannerod, 2006). (See neuroimaging evidence for the ideomotor principle in Melcher et al., 2008.) From this standpoint, conscious contents regarding ongoing action are primarily of the perceptual consequences of action (Jeannerod, 2006).

Ideomotor processing is evident in the following anecdote (mentioned in Berger et al., 2012). The television program *60 Minutes* presented a story about how, with today’s technological developments, patients can control robotic arm/limb prostheses. In the episode, the *60 Minutes* interviewer was surprised to learn that a soldier who had tragically lost his lower arm in combat could, in just a few trials, control the grasping motions of a robotic hand. The robot hand was connected to an array of electrodes attached to the muscles of the intact part of the soldier’s upper arm. The interviewer asked the soldier how, when operating the prostheses for only a few trials, it was possible to know which muscles to activate in order to have the robot enact a particular action. The soldier replied to the effect that he had no idea regarding which muscles to activate, nor what the muscles were actually doing. Rather, the soldier claimed that, to enact any action on the part of the robotic arm, all that had to be done was imagine the grasping action. This image, what Harleß in the nineteenth century called in German the *Effektbild* (in English, “the picture or image of the effect”), was somehow translated (unconsciously) into the kind of muscular activation that would normally result in a grasping action. (Additional evidence for ideomotor theory stems from *response-effect compatibility* paradigms; Kunde, 2001, in which anticipated action consequences influence how quickly one executes a given action; cf., Hubbard et al., 2011.)

These images (or mental representations) tend to mirror the real-world perceptual aspects of their outcomes (i.e., the mental representation of holding a coffee cup involves haptic and visual information, etc., Hommel, 2009). This is obvious in the case of subvocalizing (i.e., talking in one’s head). The imagery of the act is isomorphic in some sense to the act (Morsella and Bargh, 2010). Once an action outcome (e.g., grasping a cup) is selected, unconscious motor efference streams enact the action by activating the right muscles at the right time. There are, of course, also times when mental representations inhibit or cannot lead to the performance of instrumental goals. For example, though the system can represent leaping over a tall building in a single bound, limitations of the body prevent the action from occurring. Conversely, a woman holding an overheated latte can be prevented from sipping it because the “incentive systems” that are concerned with bodily needs curb her against inflicting tissue damage through one’s skeletomotor actions (Morsella, 2005; Morsella et al., 2009b). Interestingly, because of the anticipatory nature of ideomotor processing, the same stimulus (a cup) can elicit different action tendencies, with each tendency serving the same overarching goal (grasping a cup and bringing it to the mouth; see Lashley, 1942). In motor control, this is known as “motor equivalence” (Lashley, 1942). (For Lashley’s conceptualization of the role of consciousness in behavior, see Lashley, 1923.) Thus, while adaptive skeletomotor action requires integration and anticipation, it also – in some cases – requires a more elaborate form of anticipation: mental simulation/representation (Schacter and Addis, 2007).

## The echo hypothesis and nature of conscious broadcasting

Regarding the nuts and bolts of conscious broadcasting, one intriguing hypothesis stemming from observations of phenomena involving backward masking (Breitmeyer and Ögmen, 2006) and other forms of masking (e.g., object-substitution masking; Di Lollo et al., 2000) is that, for a representation to be a conscious representation, the initial modules that constructed the representation must then, in turn, receive feedback activation about that representation. An interesting aspect of consciousness is that these representations are broadcasted and available globally (Baars, 2002). Perhaps, if visual modules X and Y construct a representation for broadcast, that representation becomes conscious only after feedback activation from the broadcast returns to these two modules, much like an echo (Di Lollo et al., 2000; Fahrenfort et al., 2007). This may be because (a) this echoic (or, “reentrant”; Fahrenfort et al., 2007) processing is a necessary ingredient for the generation of consciousness (Di Lollo et al., 2000), or (b) simply because consciousness requires involvement of frontal cortex, which, after receiving the broadcast, must send top-down activation back to the modules for the representation to be conscious (Boly et al., 2011). It may also be for other, less interesting reasons, such as (c) conscious representations require a substantial amount of activation (Kinsbourne, 1996), and reentrant feedback results in this necessary increase in activation, or that (d), for a representation to be conscious, it must be activated for a long time (Lau, 2009), something that can be achieved through feedback and sustained reverberation (Hebb, 1949). Regardless of the mechanism by which feedback may be necessary for turning an unconscious representation into a conscious one, the echo hypothesis is a falsifiable proposal that can further illuminate the component processes giving rise to conscious states.

Because of the broadcasting in the conscious field, a representation is then available to more systems than just the one that produced it. Critical for a successful broadcast of any kind of information (in any system) is that there be “receivers” capable of detecting and processing the information. The nature of such receivers remains mysterious, but one can surmise that, regarding the representations at play and with respect to such receivers, these representations must possess properties that make them communicable across a wide range of brain systems, including those concerned with action control. Indeed, there is independent evidence for the notion that the representations involved in consciousness happen to be highly broadcastable (Fodor, 1983; see treatment in Godwin et al., in press). In a cyclical manner, after each broadcast, each concerned system evaluates the outputs in the field and then generates its own output, which then influences the content of the field (Baumeister and Masicampo, 2010; Morsella and Bargh, 2010). In this way, the field changes in a self-evolving manner. This is perhaps best illustrated by way of example. Imagine a student in a lecture who suddenly gets an incessant tickle in his throat and wants to cough (i.e., an “action goal” of coughing enters the conscious field). This want, however, also leads to another action goal – to not make noise in the class during the lecture. In turn, this most recent goal could lead to the willful activation of a memory of a hysterical moment from a movie to distract him from coughing, but ironically this can also lead to the action goal of suppressing a chuckle. In this way, the contents of the conscious field change over time in a multi-determined manner, with conscious contents entering it and exiting it while influencing subsequent contents, all while unconscious systems evaluate contents and contribute their own contents (Morsella and Bargh, 2010), all in the service of constraining skeletomotor action. Thus, the Jamesian stream of consciousness involves not only one conscious thought – broadcast to a plethora of receivers and leading to another conscious thought – but (a) conscious thoughts triggering unconscious processes which lead to the introduction of other conscious thoughts into the field, and (b) unconscious processes spawning their own conscious outputs, independent of field contents. Hence, the function of conscious states is not to *observe* outputs, but to allow continuous interactions among outputs and the systems that gave rise to them. Hence, perhaps it is better to compare the phenomenal field, not to a surveillance system, but to a senate (Morsella, 2005).

What we refer to as “voluntary action” occurs with all of these processes at play. The voluntary action is believed by the actor to be a function of these conscious representations (which remains possible), but it may well be that the act and the conscious representations are both determined by some other, unconscious factor (Wegner, 2002). Regardless, as mentioned above, for every voluntary act, the actor can provide through self-report an identification of a conscious content that he or she believes gave rise to the act, regardless of whether these introspections are incorrect. In the case of voluntary action, these contents tend to be anticipatory and isomorphic with action outcomes (Morsella and Bargh, 2010).

With all this in mind, it could be said that the voluntary act is, in a sense, a “loaded” action, with a heavy load of information-processing, conscious representations, and anticipatory mechanisms. This standpoint defines a voluntary action in ways more informative than the common “homuncular” definition of voluntary action – that an action is voluntary if the organism intended to do it. Our approach reveals that, unlike involuntary actions (e.g., dropping an overheated latte because of the pain withdrawal reflex), voluntary actions can be construed as a form of integrated action, which occurs when multiple action plans are co-activated and trying to influence the same skeletomotor effector. As noted by Passingham (1995, voluntary actions are special in that they can be suppressed; from present standpoint, the act of suppression (like our student suppressing his cough) is an archetypal integrated action.

## Synthesis

Building on the integration consensus, SIT proposes that conscious states integrate information-processing structures and nervous events that would otherwise be independent. According to SIT, the integration involved is primarily related to the skeletal muscle output system – where anticipatory processes play a central role – and, through a form of broadcasting, this integration controls and guides voluntary action, often via ideomotor mechanisms. Importantly, SIT is unique in its ability to explain subjective data from (a) intersensory conflicts, (b) smooth muscle conflicts, and (c) conflicts from skeletomotor conflicts (e.g., holding one’s breath and Stroop-like interference). SIT also explains why skeletal muscle is “voluntary” muscle.

Throughout the process of evolution, there has been a trend toward increased compartmentalization of function in the nervous system (Allman, 2000). In phylogeny, the introduction of new structures such as organs and tissues involves complex, often competitive interactions with previously existing ones. This problem, known as the “struggle of parts” problem (cf., Mayr, 2001), may have been a particularly formidable challenge during the evolution of something as complex as the human nervous system and could have led to various forms of “integrative solutions,” including unconscious reflexes (Sherrington, 1906; Campbell, 1993) and neural convergence (Damasio, 1989).

A fundamental assumption of our approach is that, although crosstalk between high-level action systems *could* conceivably occur without something like conscious states, such a solution was not selected in our evolutionary history. Instead, for reasons that only the happenstance process of evolution could explain (Simpson, 1949; Gould, 1977), these specific physical adaptations seem to have been selected to solve this large-scale, crosstalk problem (Morsella, 2005). Certainly, it is easy to imagine integrated actions (e.g., suppressing a chuckle) occurring without anything like conscious states, but, then again, there are many solutions to phylogenic problems that the human body did not arrive at by way of evolution. SIT aims to take an inductive and descriptive approach at understanding nervous function “as is,” and not as it (perhaps) should be. This makes SIT a *descriptive* rather than *normative* theory; and intuitions regarding how the nervous system *should* work (to be “optimal”) take a back seat to actual data revealing the manner in which it actually works (even if it is suboptimal). Hence, while some theorists have proposed that consciousness is “epiphenomenal,” (i.e., serving no function), it seems premature to arrive at such a conclusion until there is a sufficient scientific understanding about the place of consciousness in nature.

## Criticisms and alternative explanations for the current approach

SIT contrasts the actions of the skeletal muscle effector system with the actions of smooth muscle (e.g., the pupillary reflex), but it is possible that this juxtaposition could be criticized *a priori* as a false comparison because the behaviors of smooth muscle are not seen as a veritable form of action. From the point of view of such a critique, processes including the pupillary reflex, peristalsis, digestion, breathing, and other “vegetative” organismic actions should not be compared to what is commonly regarded as a typical form of action (e.g., blinking voluntarily). While we have to allow that these smooth muscle actions do not *feel* like actions, any denial of these phenomena under the category of action would exclude them, not on the basis of how an agnostic observer might see them, but because of the intuitions humans hold about the sources of their actions. If we imagine an intelligent non-human observer (e.g., an imaginary, extraterrestrial ethologist) studying the every actions humans are capable of, events such as the pupillary reflex would be worthy of being “coded” as an action just as certainly as a voluntary closing of the eyes (e.g., a wink) or an involuntary closing of the eyes (e.g., a reflexive blink; Skinner, 1953).

A second – intuitively intriguing – criticism could be that there are many aspects of conscious experience that have little or no connection to skeletal muscle plans. This criticism is rightly stated, indeed. However, in response to this criticism, it is important to distinguish the *primary* role of evolutionary adaptations from their secondary roles and current uses (Lorenz, 1963; Gould, 1977). A scientist could argue, for example, that color perception evolved for selecting fruits and detecting camouflaged prey and no sophisticated observer would counter that color perception could also be used to appreciate a painting. In fact, most people easily appreciate the idea that the color harmony of a painting is beautiful to us – at least in part – because it involves the kinds of stimuli that are of adaptive significance in another context. Similarly, SIT proposes that the *original* and *primary* function of conscious states was (and is) to integrate conflicting action plans involving skeletal muscle, not that all future and possible benefits of consciousness will be encapsulated in this single benefit.

Supramodular interaction theory proposes that conscious states involve broadcasts of the “outputs” of response systems that may conflict with the tendencies of other systems and that the outputs from response systems incessantly modulate one’s consciousness, regardless of whether there is inter-system conflict or not. Hence, there is *chronic engagement* among the systems (Morsella, 2005), assuring that no resources, time, or “intelligent homunculus” are required to decide which outputs should participate in the conscious field at a given time. That rich intelligence is embedded in the inherent structure of the apparatus, as in the case of many evolutionary products (Simpson, 1949).

It is easy to imagine a more efficient arrangement, such one that invokes conscious states only under conditions of conflict. However, chronic engagement solves the problem at a more parsimonious level. Consider that traffic lights, pool filters, and ball-return machines at bowling alleys operate and expend energy continuously, regardless of whether their function is presently needed. These systems were chosen because the cost (in this case technologically, and hence monetarily) of adding an additional detection mechanism that activates the apparatus when it is needed is greater than the benefit of what would likely be a very complex and intricate system. In this way, chronic engagement is “efficiently inefficient” in the sense that it does not require additional mechanisms to determine whether channels of crosstalk should be open or closed (Morsella, 2005). Such deceptively “inefficient” solutions can be observed in biological functions outside the nervous system, as in most biological filters (e.g., the kidneys) which continuously filter a substrate regardless of the status of the substrate.

Chronic engagement also gives rise to the oft-mentioned monitoring role of the conscious field (e.g., Angell, 1907; Norman and Shallice, 1980). However, it is misleading to characterize the field as merely supervising the outputs of response systems because the function of the field is not to *observe* outputs, but to *allow continuous interactions* among them. To build on the analogy of a senate, the senators (systems) must always be in attendance, regardless of whether they should sit quietly or debate (Morsella, 2005). Because the outputs of all the systems are always phenomenally represented (whether they are helpful or not), one experiences the subjective experience of pain even when feeling the pain is at the moment not conducive to adaptive action. And there is no way for an actor to “tell himself” that, because he needs to lose 30 pounds for a movie role, he will not experience hunger, even though the ultimately adaptive behavior for him in the modern context is to secure a role in the film.

A seeming mystery that engenders a third criticism is as follows, if conscious states are primarily for skeletomotor action, then why do conscious states continue to exist even when the skeletal muscle system is deactivated because of, for example, damage to the nervous system or a congenital disorder? In response to this criticism, one should consider the following analogy. Consider that many of today’s automobiles contain navigational systems whose primary function is to help navigate the car to one’s desired destination. With this in mind, it is conceivable that the navigational system would continue to function despite problems with, say, the transmission of the car. In a similar way, central conscious processes, whose primary function was serving skeletomotor action, can continue to function even after the peripheral structures that they are intended to serve are non-operational. It is often the case in situations where the body has been rendered ineffective, though effectors or efference generators are compromised, that the central processes that subserve the efferent processes remain intact (similar decoupling of central conscious processing from peripheral events occurs in *phantom limb*; Ramachandran, 1999). In short, consciousness is a system meant to integrate actions of systems that influence skeletal muscle, but it is not dependent on the current capacity for skeletomotor action.

We now take a moment to address alternative explanations about the phenomena we sought to explain in the selective review. First, one may argue that, in attempting to describe the function of consciousness in the nervous system, instead of proposing a framework such as SIT, it is more parsimonious to simply hypothesize that the primary role of consciousness is to suppress actions, for holding one’s breath, carrying a hot plate of food, or performing response interference tasks (e.g., the Stroop task) involves response suppression. However, this fails to account for the role of conscious states in integrated actions such as breathing faster for some reward, which requires inter-system crosstalk but no suppression.

Second, because novel skeletomotor actions tend to be executed consciously, one may argue that the function of consciousness is, not one of establishment of crosstalk for the purpose of integration, but instantiating stimulus-response relationships that are “arbitrary.” One problem with this intriguing hypothesis is that (a) it is difficult to define what constitutes an “arbitrary” mapping, (b) there are countless cases of unconscious processes that seem to involve arbitrary mappings, as in the case of motor programming (Grossberg, 1999; Rosenbaum, 2002), and (c) some non-arbitrary mappings (e.g., holding one’s breath leads to a negative subjective state) never become unconscious, despite extensive training and an inordinate amount of rehearsing the stimulus-response mappings. Moreover, unlike SIT, this hypothesis fails to explain why smooth muscle actions and intersensory conflicts are mediated unconsciously.

## Conclusion

Supramodular Interaction Theory is a framework marrying the central advancements in knowledge from the integration consensus (chiefly that consciousness is for some type of information integration) with an explanation of why “voluntary” action is described as such. This marriage leads to an explanation of the primary function (but not the only possible function) of consciousness and gets past the tautology of calling voluntary action “voluntary” because it is able to be willed. According to SIT, the integration achieved through conscious states is primarily related to the skeletal muscle output system, where anticipatory processes play a central role as in the case of ideomotor control. SIT is unique in that, while marrying consciousness to the physiological processes it subserves, it explains subjective data from (a) intersensory conflicts, (b) smooth muscle conflicts, and (c) conflict from skeletomotor conflicts (e.g., holding one’s breath). An obvious limitation of the current approach is that it sheds no light on why “subjectivity” is associated with the integrative functions these states appear to subserve. Thus, more than 40 years later, Shallice’s (1972) conclusion that consciousness is an unsolved anomaly within the scientific approach still rings true. Nevertheless, the findings presented above reveal some conceptual progress regarding the nature of consciousness in the brain. Today, one can perhaps propose that, if the heart can be conceptualized as a pump and the kidney as a filter, then consciousness can be conceptualized as a form of information broadcasting (or, more precisely, information integration). The new findings showing that subjective awareness requires reentrant (or echoic) processing present a promising direction in understanding the nature the broadcast/binding that consciousness seems to instantiate. The physical basis of the broadcasting associated with consciousness is most likely unlike anything else we currently understand.

## Conflict of Interest Statement

The authors declare that the research was conducted in the absence of any commercial or financial relationships that could be construed as a potential conflict of interest.
